# The Domain Landscape of Virus-Host Interactomes

**DOI:** 10.1155/2014/867235

**Published:** 2014-06-04

**Authors:** Lu-Lu Zheng, Chunyan Li, Jie Ping, Yanhong Zhou, Yixue Li, Pei Hao

**Affiliations:** ^1^Institute Pasteur of Shanghai, Chinese Academy of Sciences, 320 Yueyang Road, Shanghai 200031, China; ^2^Hubei Bioinformatics and Molecular Imaging Key Laboratory, Huazhong University of Science and Technology, 1037 Luoyu Road, Wuhan 430074, China; ^3^Shanghai Center for Bioinformation Technology, 1278 Keyuan Road, Shanghai 201203, China; ^4^Key Laboratory of Systems Biology, Shanghai Institutes for Biological Sciences, Chinese Academy of Sciences, 320 Yueyang Road, Shanghai 200031, China

## Abstract

Viral infections result in millions of deaths in the world today. A thorough analysis of virus-host interactomes may reveal insights into viral infection and pathogenic strategies. In this study, we presented a landscape of virus-host interactomes based on protein domain interaction. Compared to the analysis at protein level, this domain-domain interactome provided a unique abstraction of protein-protein interactome. Through comparisons among DNA, RNA, and retrotranscribing viruses, we identified a core of human domains, that viruses used to hijack the cellular machinery and evade the immune system, which might be promising antiviral drug targets. We showed that viruses preferentially interacted with host hub and bottleneck domains, and the degree and betweenness centrality among three categories of viruses are significantly different. Further analysis at functional level highlighted that different viruses perturbed the host cellular molecular network by common and unique strategies. Most importantly, we creatively proposed a viral disease network among viral domains, human domains and the corresponding diseases, which uncovered several unknown virus-disease relationships that needed further verification. Overall, it is expected that the findings will help to deeply understand the viral infection and contribute to the development of antiviral therapy.

## 1. Introduction

Viral infections result in millions of deaths each year. AIDS has become one of the leading killers worldwide and the influenza has always been a headache of public health organizations. Governments around the world annually invest billions of dollars to investigate the mechanism of viral infections, potential targets for treatment, and innovative vaccines. It is widely accepted that viral infection and pathogenesis mainly depend on their ability to interact with human proteins through a complex network of protein-protein interactions (PPIs). For humans, eukaryotic cells express a large group of proteins to develop normal function through a highly connected and two-side network, which exhibits robustness against random attack and a high sensitivity to targeted subversion [1, 2]. The smart virus takes advantage of this characteristic to evolve an efficient strategy of hijacking central proteins and interfering with the regulated network, aiming to complete its own life cycle [3, 4]. This perturbation often damages the host cellular networ, and thus causes severe diseases, like the occurrence of cancers [5, 6]. Rozenblatt-Rosen et al. have confirmed the hypothesis that genomic variations and virus proteins may lead to cancer in similar ways, such as Notch signalling and apoptosis, by examining systematically host interactome network perturbations caused by DNA tumor viruses [7]. A thorough analysis of virus-host interactomes may reveal insights into viral infection and pathogenic strategies and help identify novel drug targets [8] and decipher the molecular aetiology of some complex diseases [9]. With the help of high-throughput experiments [10–12] such as yeast-two hybrid screens or literature mining, researchers have collected many virus-host PPIs, generating invaluable virus-host PPI databases [13, 14] and tried to provide a global view of human cellular processes controlled by viruses [8]. However, we found that this global analysis ignored the structural details of individual proteins and their interaction interfaces, which limited our comprehensive understanding.

It is well established that many PPIs are mediated by protein domain pairs. The domain, a stable part of protein structure, evolves and functions independently. The domain is usually used to combine with other domains to form a multidomains protein [15], which functions through interacting with domains from other proteins. Itzhaki et al. [16, 17] indicated that domain-domain interactions (DDIs) actually reflected an evolutionary conservation; that is, the same DDI might occur in different organisms and many PPIs might also be attributed to a limited set of DDIs. DDIs underlying herpes virus-human PPI networks have showed that viral domains tend to interact with human hub domains [17]. Therefore, DDIs, as the building blocks of PPIs, provide an attractive abstraction of protein network and capture the dynamics of interactions in the cellular system.

In this study, we developed an integrated pipeline to construct a virus-host interactome based on protein domain pair, where we hoped to present novel insights that might not be provided in the binary protein interaction networks. Therefore, we performed topological and functional analysis of this interactome. Moreover, we attempted to map somatic mutations to human domains and gain novel associations between viruses and diseases.

## 2. Materials and Methods

### 2.1. Used Dataset

We downloaded literature-curated binary PPIs in July 2012, from ten public databases: the Biomolecular Interaction Network Database (BIND) [18], the Database of Interaction Proteins (DIP) [19], the Human Protein Reference Database (HPRD) [20], IntAct [21], the Molecular INTeratcion database (MINT) [22], Virus-Host Network (VirHostNet) [14], HIV-1, Human Protein Interaction Database [23], the Biological General Repository for Interaction Datasets (BioGrid) [24], InnateDB [25], and Pathogen-Host Interaction Search Tool (PHISTO) [26] (Table 1). We collected intravirus, virus-host, and intrahost PPIs from these databases and only physical PPIs were remained. Since not all databases used the uniform IDs, such as Uniprot [27] or GenBank [28], we removed redundant PPIs based on the protein sequences in the same species. The protein sequences were retrieved from Uniprot, GenBank, Ensembl [29], and DIP, according to each database's ID type. There were 135,231 intrahost PPIs among 44,078 proteins, 13,058 virus-host PPIs between 674 viral proteins from 94 viruses, and 2,388 host proteins (See Supplementary Table 1 in Supplementary Material available online at http://dx.doi.org/10.1155/2014/867235).

We downloaded the DDI dataset from Integrated Domain-Domain Interaction Database (IDDI) [30], which included three structure-derived DDI datasets (3DID [31], iPfam [32], and PInS [33]) and twenty computationally predicted DDI datasets. It has developed a novel scoring scheme to measure the reliability of each DDI by considering their prediction scores, independencies among the twenty datasets, and the confidence levels of each prediction method in the datasets. It currently contains 204,705 unique DDIs between 7,351 distinct Pfam domains, where 6,768 interactions are from 3D structure-derived datasets.

### 2.2. Virus-Host Interactome Network Based on Domain Interactions

All proteins in the PPI dataset were scanned by the Pfam scan utility and HMMER 3.0 with default parameters against Pfam-A models obtained from Pfam (v.26.0) [34]. Then, we mapped the PPIs to DDIs according to the following rule: if two interacting proteins contained domains documented as interacting (e.g., DDIs from IDDI database), where one domain located in one protein and the other in the interacting partner, the interaction of these two proteins could be attributed to this domain pair.

We integrated all the structure-derived DDIs and top 30% of predicted DDIs in consideration of the reliability and quantity. Additionally, DDIs were further filtered to exclude those domain pairs that were reported as non-interacting by the Negatome database [35]. Therefore, there are 9,598 intrahost DDIs among 2,084 domains, 1851 intravirus DDIs among 839 domains, and 269 virus-host DDIs between 87 viral domains from 49 viruses and 144 host domains (Supplementary Table 1).

### 2.3. Topological Analysis in the Host DDI Network

The degree or connectivity of one domain node in a graph is the number of edges that are linked to this domain node, which stands for a local centrality measure. The betweenness of one domain node *v* in a graph is a global centrality measure which is defined by the number of shortest paths going through this node between any pairs and normalized by twice the total number of protein pairs in the graph (*n*∗(*n* − 1)/2, supposing there are *n* nodes in the graph). The equation used to calculate the betweenness centrality of the node *v*, *b*(*v*), is as follows:

(1) $$\begin{array}{c} b\left(v\right)=\frac{1}{2\times \left(\left(n\times \left(n-1\right)\right)/2\right)}\times \underset{i,j,v\in V,i\neq j\neq v}{\sum }\frac{g_{ij}\left(v\right)}{g_{ij}}, \end{array}$$

where *g*_*ij*_ is the number of shortest paths going from node *i* to *j* and *g*_*ij*_(*v*) is the number of shortest paths from *i* to *j* that pass through the node *v*. A domain with high degree or betweenness centrality is characteristic of a hub or bottleneck in an interaction network and often is critical to this network [36]. In the intrahost DDI network, all domains could be divided into two parts: one that would be targeted by viruses (targeted domains) and the other that would not be targeted (nontargeted domains). We plotted distributions of degrees and betweenness centralities for these two types of domains, respectively. If the distributions of targeted domains were more biased towards high degree and betweenness centrality domains than the distribution for nontargeted domains, then we hypothesized that viruses had evolved to interact with hub and bottleneck domains in the host DDI network.

The average shortest path length, also called characteristic path length, is defined as the average of all the shortest path lengths between the nodes in the graph. It measures the efficiency of information transport on a network. The local clustering coefficient measures the probability that the adjacent vertices of a vertex are connected, and the clustering coefficient of a network is the average local clustering coefficient of all vertices of the network. Simply, the local clustering coefficient of a node *v* is calculated as

(2) $$\begin{array}{c} cc=\frac{2\left|E\right|}{k\left(k-1\right)}, \end{array}$$

where *E* is the edges between neighbors of *v* and *k* is the degree of *v*. Thus, a large clustering coefficient means that neighbors of a node tend to cluster together. We used the R package igraph [37] to compute these network topological parameters. Self-interactions were not taken into account in these interactions.

### 2.4. Functional Analysis of Domain Sets

To analyze the functional impact of host domains targeted by viruses, we conducted an enrichment analysis of GO [38] terms. The mapping of Pfam-A domains to their GO functions is obtained from pfam2go in the GO website (http://www.geneontology.org/external2go/pfam2go). Using all Pfam-A domains of the human's proteome as universe, we sought to find enriched functional terms associated with viral infection and infection mechanisms using R and Bioconductors topGO package [39]. We used the weight algorithm of topGO, which eliminated local similarities and dependencies between GO terms in the GO graph during the analysis. Statistical significance level was set to 0.05. All three GO terms (biological process, molecular function, and cellular component) were scanned to identify the terms having significant association with each studied host domain set.

### 2.5. Compiling a Comprehensive List of Disease, Disease-Associated Genes (Domains), and Mutations

The Ensembl variation database imports variation data (SNPs, CNVs, genotypes, phenotypes, etc.) from a variety of sources (e.g., dbSNP) [29]. For humans, it also integrates variants from HGMD-PUBLIC [40], OMIM [41], and COSMIC [42] datasets, in which variants are linked to human genes and the corresponding diseases. It includes germline variants and somatic variants, and the latter are all from COSMIC. Moreover, the Ensembl classify the variants into different classes and calculate the predicted consequence(s) of each amino acid substitution on each protein by using PolyPhen-2 program, where score 1 means the most damaging [43]. We extracted missense (SNPs, in-frame short inserts and deletions) and truncating (frameshift indels and stop gain) mutations with related proteins and diseases from the Ensembl variation 69 dataset. The protein IDs from Ensembl to UniprotKB were converted based on the protein sequences. And mutations on the proteins that could not be converted were discarded. For proteins that contained nonsynonymous somatic mutations, we generated a cumulative somatic mutation score for each protein after normalizing scores for protein length. All proteins with at least one nonsynonymous somatic mutation were ranked by the normalized cumulative scores, where TP53 ranked the highest. Statistical significance assessment of overlap between gene sets was performed with Fisher's exact test. And gene sets' GO pathway analysis was performed by DAVID [44].

Likewise, by mapping all disease-associated mutations to the corresponding domains after Pfam scan utility scanning, a total of 210,887 mutations, including 177,493 missense and 33,394 truncating mutations, were obtained in 3,906 domains associated with 3,531 clinically distinct disorders. Insertions and deletions were mapped to domains using the starting position of the mutations. We also ranked these domains according to their normalized cumulative score, where the domain was normalized by dividing by the cumulative length of all occurrences of the domain within proteins, with VHL, NOD, and P53 having the highest scores. Among these, 118 domains from 220 proteins with 3,832 mutations were targeted by viruses.

## 3. Results

**Figure 1:**
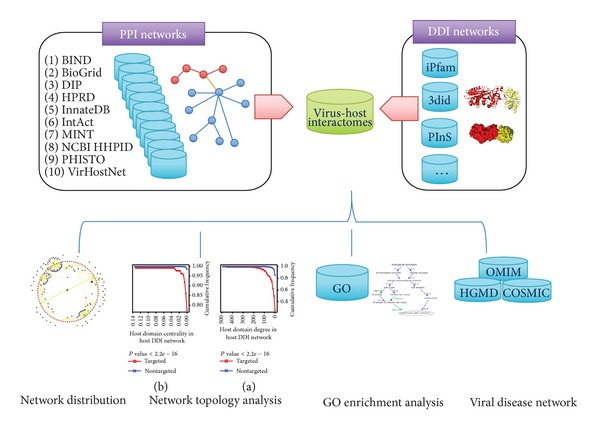
Workflow of the construction and analysis of virus-host interactomes.

We built the virus-host interactomes by screening domain interactions between virus-host PPIs, and then we studied the network distribution, performed topological and function analysis, and speculated the association between viruses and diseases (Figure 1). According to the type of genome and the method of replication, we partitioned all the viruses into three categories: DNA viruses, RNA viruses, and retrotranscribing viruses, as well as different families and genera according to the taxonomy database annotation from NCBI. Noticeably, delta virus was excluded because it belongs to a satellite virus, which is not divided into DNA, RNA, or retrotranscribing viruses by the taxonomy database.  Table 2 shows the statistics of virus-host interactome according to three viral categories.

### 3.1. Domain and DDI Distributions

First, we examined the distribution of domains and DDIs in virus-host interactomes.  Table 3 lists viral domains occurring in at least three species and host domains targeted by at least three species, as well as virus-host DDIs occurring in at least three species. For viral domains, it showed that most domains were unique, and no domain was overlapped among three categories of viruses, indicating that viruses tended to use their own protein domains to mediate cross-species PPIs. The most frequent domains were PF00527 and PF00098, which were conserved among five species from Papillomaviridae and Retroviridae family, respectively. For host domains, we observed that six domains were targeted by all three categories of viruses: one RNA recognition motif, two kinase phosphorylation-related, and three immunity-related domains (Table 3). It is not surprising that viruses target these domains. For example, PF00069 (protein kinase domain) is involved in a process called phosphorylation and functions as an on/off switch for many basic cellular processes. When viruses invade the host, they have to modify cell physiology, metabolic pathway, and regulatory networks to gain control of fundamental processes, such as transcription, cell cycle, and apoptosis. A well-characterized example was provided by the HIV-1 Tat protein, which used the phosphorylation of human CDK9 to stabilize the interactions between Tat (PF00539, transactivating regulatory protein Tat) and CDK9 (PF00069) and then to help promote productive elongation of HIV mRNA [45, 46]. Consequently, CDK9 was required for HIV to hijack host transcription machinery during its replication, and its inhibitors might become novel and specific antiretroviral agents [47, 48]. For virus-host DDIs, it revealed that only a few were the same among different viral species, and so they were also more likely to be unique (Table 3).

Then, we put the virus-host DDIs in the context of human interactome and investigated that some conserved DDIs not only mediate interactions within host but also in the virus-host interface.  Table 4 lists ten of them, which appeared in human interactome most frequently. We identified 35, 4, and 41 conserved DDIs for DNA, RNA, and retrotranscribing viruses, respectively, but no overlapped DDIs existed among them. In fact, viral domains probably competed with human domains to interact with their human domain partners. For instance, Epstein-Barr virus early antigen protein BHRF1 acts as a host B-cell leukemia/lymphoma 2 (Bcl-2) homolog and may competitively interact with the human protein VRK2, which is involved in preventing premature death of the host cell during virus production [49]. These two interactions could be attributed to the PF00452 (apoptosis regulator proteins, Bcl-2 family) found in both virus and human, and the PF00069 in human. A little surprisingly, although some DDIs such as PF00069-PF00069 were derived from the most intrahost PPIs, only one virus evolved the ability to use it to cross the interspecies barrier. In addition, the numbers of viral domains shared with host ones were 16, 2, and 5 for DNA, RNA, and retrotranscribing viruses, respectively. Along with the evolution, large DNA viruses capture DNA sequences from their host that encodes complex functional domains and integrates them into their own genomes [50], and then DNA viruses acquire the ability to finely tune the metabolism of infected cells by competitive interactions. It seems like this strategy allows some DNA viruses not to affect host cellular networks immediately but induce chronic infections at last. We also noticed that retrotranscribing viruses owned more conserved DDIs than RNA viruses because of the additional retrotranscribing process and the research bias in HIV.

### 3.2. Viruses Target Human Hub and Bottleneck Domains

**Figure 2:**
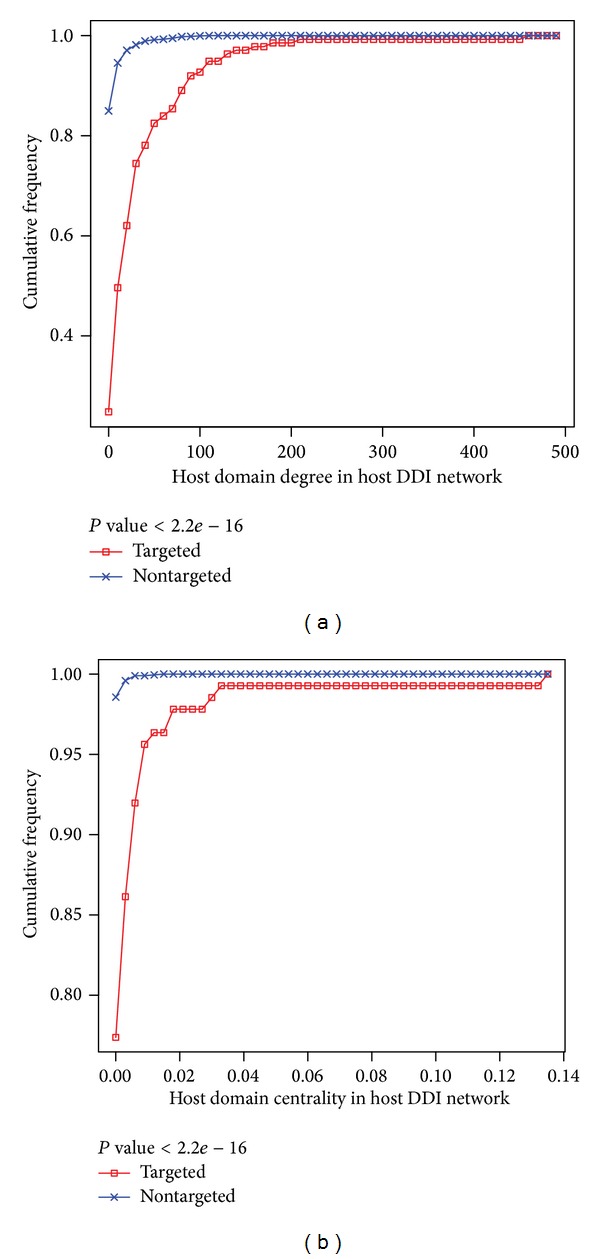
Cumulative degree and betweenness centrality distributions. Host domains that are targeted by viruses (targeted) have a higher degree and betweenness centrality than the domains that are not targeted by viruses (nontargeted): (a) degree distribution and (b) betweenness centrality distribution. These findings are statistically significant by Wilcoxon rank sum test. The cumulative frequency at a particular value of degree or centrality is the percent of domains whose degree or centrality are less than this particular value.

Many studies have showed that viral proteins tend to interact preferentially with hub and bottleneck proteins in the human interactome network [8, 26, 51, 52]. This is consistent with the parsimonious use of viral genetic materials to control the host biological networks effectively. Similar results were also observed in the virus-host DDI network. As Figure 2 shows, compared with nontarget host domains, viral target domains are distinct in two ways: it is more significantly connected to other host domains; it is in a more central position in the context of human interactome.

**Figure 3:**
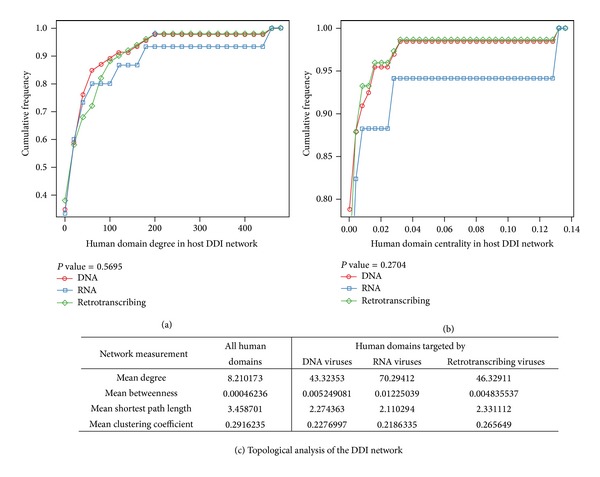
Cumulative degree and betweenness centrality distributions among DNA, RNA, and retrotranscribing viruses. Host domains that are targeted by DNA, RNA, and retrotranscribing viruses, respectively, have an approximate degree and betweenness centrality: (a) degree distribution and (b) betweenness centrality distribution. These findings are statistically significant by Fligner-Killeen (median) test. The cumulative frequency at a particular value of degree or centrality is the percent of domains whose degree or centrality are less than this particular value. (c) Topological analysis of the human domains and of the human domains targeted by viruses in the human interactome. The mean degree, the mean betweenness centrality, the mean shortest path length, and mean clustering coefficient are given first for all the human domains, then for the human domains targeted by DNA, RNA, and retrotranscribing viruses.

For the viral target domains, we examined top ten hub and bottleneck domains with highest degrees and betweenness centralities (Table 5). All the domains were involved in some fundamental functions, such as transcriptional regulation or signal transduction. Nine of them were overlapped between hub and bottleneck domains. For example, protein kinase domain/RNA recognition motif ranked first/third both in the degree and betweenness centrality and were targeted by all three groups of viruses, implying these two domains' indispensability. As described before, the protein kinase domain is a structurally conserved protein domain containing the catalytic function of protein kinases, and the human genome encodes about 518 protein kinase genes [53]. The RNA-recognition motif (RRM) is one of the most abundant protein domains in eukaryotes. It has been estimated that up to 1% of human genes encode proteins that contain one or more RRMs [54]. RRM-containing proteins are involved in many posttranscriptional gene expression processes (e.g., mRNA and rRNA processing, RNA export and stability) [55]. Viruses such as dengue virus, vaccinia virus, and HIV-1 have evolved different mechanisms to bind host RRM-containing proteins to facilitate their genomes replication or mRNA translation [56–58]. We further compared degree/betweenness centrality distributions of the host domains targeted by DNA, RNA, and retrotranscribing viruses (Figure 3). Average degrees of viral target domains were five to eight times higher than the average degree of the human interactome, and average betweenness was 10 to 26 times higher. Other deeper network analysis showed that the average shortest length of viral target domains was lower than the human interactome, while the clustering coefficient was nearly identical. But no significant difference was observed between the three categories of viruses (Figure 3). Therefore, as a general hallmark, all viruses tended to interact with targets and cellular pathways that were highly interconnected and central, as well as relatively close to each other in order to amplify their effects on host cellular system.

**Figure 4:**
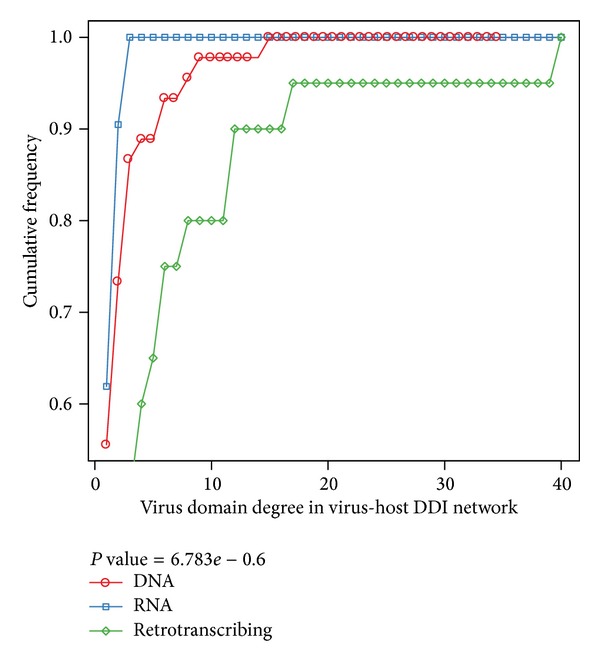
Cumulative degree distribution of viral domains among DNA, RNA, and retrotranscribing viruses. In the virus-host DDI network, we compared the connectivity of viral domains from DNA, RNA, and retrotranscribing viruses. The finding is statistically significant by Fligner-Killeen (median) test. The cumulative frequency at a particular value of degree is the percent of domains whose degree are less than this particular value.

In addition, we compared the degree of viral domains in virus-host DDI network.  Figure 4 shows that there are significant differences. The degrees of viral domains were heterogeneous. Retrotranscribing viruses had the most host targets, and followed by DNA viruses and RNA viruses. The largest connectivity of retrotranscribing virus domain (PF00539, Tat) was 40, while it was only 3 and 15 for RNA (e.g., PF00073, picornavirus capsid protein) and DNA virus domain (PF00226, DnaJ domain), respectively. It is expected that the connectivity of retrotranscribing viruses is much higher than the other two. On one hand, retrotranscribing viruses have small genomes encoding a few domains. They have to hit multiple cellular targets to perform a sufficient number of tasks during the entire viral life cycle. A good example of such multitasking domains is provided by PF00469 (Nef) of HIV-1, which downregulates the expression of the surface MHC-I molecules, CD4, and interleukin-2 receptor [59, 60]. On the other hand, many researches put so much focus on HIV, which belongs to retrotranscribing viruses. Therefore, there were much more HIV-human PPI data, which contribute to much more DDI relations.

### 3.3. Functions Enriched in Human Domains Interacting with Viruses

We divided host domains into the following sets: domains targeted by any virus (overall set), domains by DNA viruses (DNA set), domains by RNA viruses (RNA set), and domains by retrotranscribing viruses (retro set), as well as domains by different families, genera, and species. We computed overrepresented GO terms of host domains in above sets. Overall, we found 19 unique enriched GO (Biological Process) terms (Table 6). All enriched GO terms for each set are available in Supplementary Table 2 for further analysis. We concluded that viruses adapted to attack domains generally involved in host's transcription, cell cycle, apoptosis, and immunity modulation. They manipulated host's transcriptional machinery to proliferate during infection. Meantime, they needed to evade or suppress host's immunity defense. For example, we noticed that “immune response” and “antigen processing and presentation” were targeted by multiple species, multiple genera, multiple families, and even multiple categories of viruses (Table 6). Indeed, when viral pathogens enter into the human body, the human cells recognize their invasion through pattern-recognition receptors (PRRs) [61] and mount strong antiviral responses, including innate and acquired immunity. However, viral pathogens evolve several methods to elude host immune responses. From Table 3, we know that PF00129 and PF06623 are targeted by all three categories of viruses, such as EBV, HIV-1, and influenza A virus. These two domains are both found in the region of the MHC class I molecules, which can present foreign antigens such as viral peptides to T cells responsible for cell-mediated immune responses, for example, HLA-B*27:05 (MHC class I gene) is able to recognize RRIYDLIEL epitope in the EBNA-3C of EBV and SRYWAIRTR epitope in the nucleoprotein of influenza A viruses [62]. However, the killer Ig-like receptor (KIR)3DL1 cannot recognize EBV-HLA-B*27:05 complex [62], and if the substitution of R2G or W4Y or W4F in the wild-type epitope SRYWAIRTR of influenza virus occurs, it will result in a substantial reduction of recognition to cytotoxic T lymphocytes (CTLs) [62, 63]. Unlike EBV and influenza, HIV-1 has developed a different mechanism to evade host defense, for example, by downregulating the expression of surface MHC-I molecules [59].

Our analysis also highlighted an interesting mechanism “double-strand break repair via nonhomologous end joining” (Table 6), which was enriched by DNA viruses and retrotranscribing viruses. In eukaryotic cells, cells usually possess two major pathways to repair double-strand DNA breaks: homologous recombination (HR) and nonhomologous end joining (NHEJ) [64, 65]. Therefore, the DNA repair machinery acts as an intrinsic cellular defense. At the same time, it recognizes viral genetic materials as damaged DNA and restricts viral proliferation [65].* In vitro* assay shows DNA-dependent protein kinase (DNA-PK), which plays an important role in NHEJ [65]. Our dataset demonstrated that SV40-LTag could interact with human Ku70 protein, whose dimer acted as regulatory subunit of the DNA-PK complex through PF00226 (DnaJ domain) targeting PF02735 (Ku70/Ku80 beta-barrel domain) or PF03730 (Ku70/Ku80 C-terminal arm), which indicated that SV40 might benefit from the cellular DNA-damage signalling. Much remains to be learned about how SV40 infection activates DNA-damage signalling and uses it to facilitate viral propagation. HIV-1 evolves a similar strategy to protect itself and to promote its replication. Its integrase (IN) interacts with N-terminal part of Ku70 (PF02735) to protect IN from the Lys-linked polyubiquitination proteasomal pathway and to assist IN integration activity during viral assembly, independent of Ku70/80 heterodimerization [66]. Other GO functions, such as “protein phosphorylation,” “regulation of transcription, DNA-dependent,” and “regulation of apoptotic process,” are also manipulated by at least 2 species or families (Table 6), uncovering important pathways in the progression of viral infection.

### 3.4. Viral Disease Network

Complex biological systems and cellular networks may underlie most relationships from genotype to phenotype. Understanding genotype-phenotype relationships requires that phenotypes be viewed as manifestations of network properties, rather than simply as the result of individual gene variations. Some studies have proved that missense point mutations and in-frame short indels associated with the corresponding human disorders are enriched on the interaction interfaces of proteins [67]. The idea that abnormal alteration (disruption or enhancement) of specific protein interactions can lead to human diseases complements canonical gene loss/perturbation models and provides new clues on mechanisms underlying human diseases [67]. Interestingly, if a virus targets a human disease susceptibility protein competitively, especially an interaction interface, what will happen? We can presume that this virus may cause the similar diseases like some specific mutations in this protein. This can be used to uncover new disease phenotype associated with infection viruses with genomic approaches.

**Figure 5:**
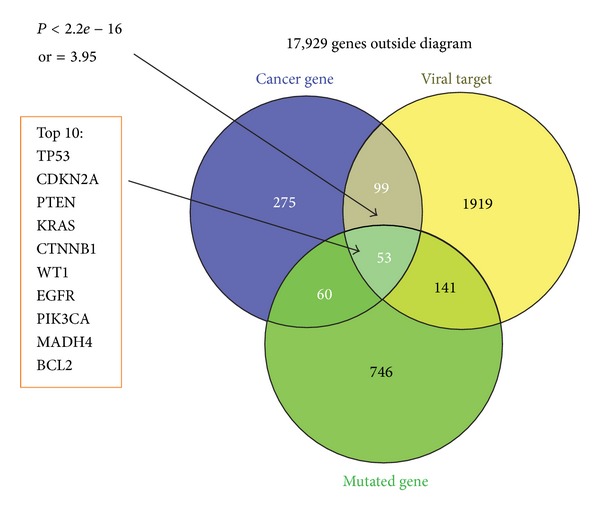
Viral targets enriched in cancer-related genes. Venn diagram of overlaps among viral target proteins, cancer genes, and a set of top 1000 genes through somatic mutation analysis. *P* value is based on Fisher's exact test.

To systematically investigate the extent to which viral proteins globally targeted host proteins causally implicated in cancer, we first compared viral target proteins against a gold standard set of 487 high-confidence causal human cancer genes in the COSMIC Classic Genes set identified by Cancer Gene Census [42]. Viral targets were found to be enriched among this COSMIC Classic Genes significantly (*P* < 2.2*e* − 16, odds ratio = 3.95; Figure 5). In another way, we compiled ~310,000 non-synonymous somatic mutations for ~17,000 human genes. Depending on the PolyPhen-2′s variation effect score for each nonsynonymous mutation in each gene, we got a normalized cumulative score for each protein. We picked up the top protein from those ones belonging to the same gene according to ranking. Then we selected the top 1000 genes to compare with our viral targets and COSMIC classic genes (Figure 5). It's shown that the cross section of three parts covered 53 genes, in which top 10 were included in the top 20 of ranked genes (Supplementary Table 3). Pathway analysis of the 53 genes revealed that 20 genes could be implicated in the GO pathway linked to “regulation of apoptosis” (GOTERM BP FAT, *P*_adj_ = 4.8 × 10^−9^). Other GO terms also covered plausible contributors to cancer pathogenesis, such as “regulation of cell proliferation.” Consequently, many human cancers might not only arise from mutations of disease susceptibility genes, but also from viral infections, which just used these disease susceptibility genes as their direct or indirect targets.

Further, we constructed a domain-cancer network with 3,906 domains and 3,531 relevant diseases by mapping variations to domains on the corresponding disease proteins. And these domains containing at least one nonsynonymous somatic mutations were also ranked according to their normalized cumulative scores (Supplementary Table 4). We examined that viral target domains were enriched in cancer-related domains (*P* = 3.015 × 10^−9^ with Wilcoxon rank sum test). Undoubtedly, viruses tended to target cancer-related domains. Compared with overall disease-related domains, it was shown that the hub and/bottleneck domains (top 10% of nodes with highest degrees and betweenness centralities) ranked higher by their normalized cumulative score (*P* = 3.817 × 10^−10^ and *P* < 2.2 × 10^−16^ with Wilcoxon rank sum test, resp.), meaning that hub or bottleneck domains might preferentially relate with human cancers, and cancer-related domains equally had more interaction partners than noncancer domains in DDI networks.

**Figure 6:**
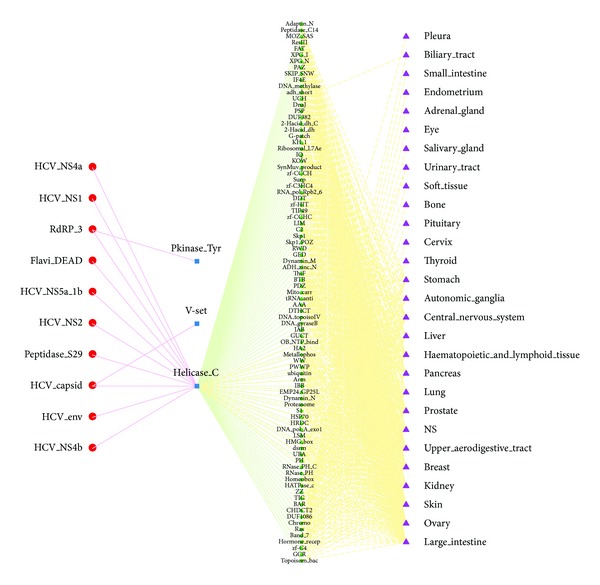
Virally implicated diseases network for HCV. Red dot: HCV domains; blue square: HCV target domains; green diamond: host domains regulated by viral targets; purple triangle: tumor sites.

A striking aspect was to link viruses, human genes/domains, and the corresponding diseases to determine whether the relationships could interpret known pathogenic mechanisms and to predict novel potential associations between viruses and virally implicated diseases. Here, we focused on the construction of disease network between HCV and somatic mutations that led to cancer in different tissues (Figure 6). 10 HCV domains mainly targeted Helicase_C domain, which was a conserved C-terminal domain on helicases and helicase related proteins, and was also responsible for dsRNA recognition. We put these three viral targets in the context of human interactome and found that Helicase_C was able to bind other 87 host domains related with cancer in 28 tissues. It was noticed that many host domains such as Helicase_C and FAT were associated with liver cancer. As we all know, HCV is a hepatitis virus and can result in permanent liver damage and hepatocellular carcinoma (HCC). At the same time, HCV infection has also been associated with numerous extrahepatic manifestations, including renal, dermatologic, hematologic, and rheumatologic systems [68]. Our results suggested that HCV might be connected to several cancers that had not been previously reported (Figure 6).

## 4. Conclusion

In conclusion, we presented a global landscape of virus-host interactomes from a domain-centric view. In contrast to the analysis at protein level, our virus-host DDIs, considered as the building blocks of interactomes, provided an attractive abstraction of PPI networks and reduced the bias of the analysis in PPI networks because the number of retrotranscribing viruses accounted for more than 70% in PPI datasets, whereas it dropped to less than 50% in DDIs. In the virus-host interactomes, we observed that viruses use unique domains to interact the same host partners with fundamental functions. Meanwhile, viruses used conserved DDIs occurring in host interactomes to mediate the interspecies interaction. On the topological side, results showed that viruses preferentially interacted with hub and bottleneck domains in the context of host interactomes, which was consistent with PPI network. The degree and betweenness of three categories of viruses were significantly different. On the functional side, we found that viruses perturbed the host cellular network by both common and unique strategies. Most importantly, we linked the viral infection and caner and then observed that genomic variation and viral protein interaction might alter local and global properties of host cellular networks to induce pathological states in the similar way. Then, we constructed a virus-disease network to uncover several cancers that had not been previously associated with viral infections. However, our findings should be interpreted with caution, since virus-host interactomes were still a little limited. First, many proteins in virus-host PPI network had no domain assigned, because Pfam-A model did not cover the complete protein domain universe. Second, we used a putative strategy to map PPIs to DDIs, since very few virus-host PPIs were obtained crystallographically. Overall, our results will help deeply to identify molecular mechanism associated with viral infection and contribute to better strategies for antiviral therapy.

## Supplementary Material

The supplementary materials include detailed statistics of intraspecies and interspecies of PPIs and DDIs (Supplementary Table 1). The supplementary materials also include GO enrichment results for different host domain sets by topGO, including BP, MF and CC. We computed enriched GO terms for each species, genera, family, type and all of viruses, and “1” in each cell means the corresponding GO term is enriched (P<0.05), otherwise 0 (Supplementary Table 2). Supplementary Table 3 and 4 are ranked gene and domain lists according to their cumulative normalized scores.

## Figures and Tables

**Table 1:** Statistics of intraviral, intrahost, and interspecies PPIs in databases.

Database	Number of PPIs	Number of proteins	Number of intrahost PPIs	Number of intravirus PPIs	Number of interspecies PPIs
BIND	20479	14098	5364	684	1109
BioGrid	349696	43462	62805	7	569
DIP	70127	24351	2866	164	405
HPRD	39198	9673	39198	0	0
InnateDB	8359	3862	5959	0	0
IntAct	234264	59238	41315	582	570
MINT	26239	10741	21686	445	1248
NCBI_HHPID	2582	1462	0	0	2582
PHISTO	14928	3253	0	0	14928
VirHostNet	11426	5602	6610	1650	3113

**Table 2:** Statistics of intravirus, virus-host PPIs, and DDIs.

	DNA virus	RNA virus	Retrotranscribing virus
Number of PPIs	1188	1046	10820
Number of viral proteins	266	101	303
Number of human proteins	615	579	1633
Number of DDIs	107	31	130
Number of viral domains	45	21	20
Number of human domains	68	18	85

**Table 3:** Distributions of domains and DDIs.

Virus	Number of species^∗a^	Number of genera	Number of families	Number of types	Description
PF00527	5	3	1	1	E7 protein, early protein
PF00098	5	3	1	1	Zinc knuckle
PF00511	4	3	1	1	E2 (early) protein, C terminal
PF00508	4	3	1	1	E2 (early) protein, N terminal
PF00073	3	1	1	1	Picornavirus capsid protein
PF00423	3	2	1	1	Haemagglutinin-neuraminidase

Host	Number of species^∗a^	Number of genera	Number of families	Number of types	Description

PF00069	10	7	6	3	Protein kinase domain
PF07686	7	5	5	3	Immunoglobulin V-set domain
PF00018	6	4	3	2	SH3 domain
PF00129	6	5	3	3	Class I histocompatibility antigen, domains alpha 1 and 2
PF02319	5	3	2	2	E2F/DP family winged-helix DNA-binding domain
PF07716	5	4	2	2	Basic region leucine zipper
PF07714	5	4	3	3	Protein tyrosine kinase
PF00076	5	3	3	3	RNA recognition motif
PF06623	4	3	3	3	MHC_I C-terminus
PF00134	3	2	2	2	Cyclin, N-terminal domain
PF00397	3	3	1	1	WW domain
PF00870	3	2	2	2	P53 DNA-binding domain
PF00240	3	2	2	2	Ubiquitin family
PF03066	3	2	2	2	Nucleoplasmin
PF00017	3	2	1	1	SH2 domain
PF00170	3	3	3	2	bZIP transcription factor
PF00605	3	2	2	2	Interferon regulatory factor transcription factor

Virus-host DDI	Number of species^∗a^	Number of genera	Number of families	Number of types	Number of intrahosts

PF00527-PF02319	4	2	1	1	0
PF00508-PF07716	4	3	1	1	0
PF00511-PF07716	4	3	1	1	0
PF00098-PF00397	3	3	1	1	1
PF00098-PF00069	3	1	1	1	0

**Table 4:** Conserved DDIs between interspecies and intrahost.

Virus-host DDI	Number of species	Number of genera	Number of families	Number of types	Number of intrahosts
PF00069-PF00069	1	1	1	1	390
PF00017-PF07714	1	1	1	1	253
PF00018-PF00018	2	1	1	1	203
PF00017-PF00017	1	1	1	1	203
PF00018-PF00017	1	1	1	1	176
PF07714-PF00017	1	1	1	1	170
PF00017-PF00018	2	1	1	1	150
PF07714-PF07714	1	1	1	1	145
PF00018-PF07714	1	1	1	1	144
PF07714-PF00018	2	1	1	1	102

**Table 5:** Top 10 hub and bottleneck domains.

Pfam	Degree	Description	Pfam	Betweenness centrality	Description
PF00069	464	Protein kinase domain	PF00069	0.136	Protein kinase domain
PF00400	214	WD domain, G-beta repeat	PF00400	0.035	WD domain, G-beta repeat
PF00076	186	RNA recognition motif (RNP domain)	PF00076	0.031	RNA recognition motif (RNP domain)
PF00018	166	SH3 domain	PF00018	0.020	SH3 domain
PF00105	144	Zinc finger, C4 type (two domains)	PF00240	0.018	Ubiquitin family
PF00271	133	Helicase conserved C-terminal domain	PF00071	0.012	Ras family
PF00240	133	Ubiquitin family	PF00046	0.011	Homeobox domain
PF00169	117	Pleckstrin homology domain	PF00271	0.011	Helicase conserved C-terminal domain
PF00104	116	Ligand-binding domain of nuclear hormone receptor	PF00105	0.010	Zinc finger, C4 type (two domains)
PF00071	114	Ras family	PF00169	0.010	Pleckstrin homology domain

**Table 6:** Enriched GO (biological process) terms of domain sets.

GO ID	GO term	Number of species	Number of genera	Number of families	Number of types	Overall
GO:0006303	Double-strand break repair via nonhomologous end joining	2	2	2	2	1
GO:0006351	Transcription, DNA-dependent	2	1	1	1	1
GO:0006468	Protein phosphorylation	8	4	2	1	0
GO:0006355	Regulation of transcription, DNA-dependent	4	2	2	1	0
GO:0007165	Signal transduction	1	0	0	0	0
GO:0045892	Negative regulation of transcription, DNA-dependent	1	0	0	0	0
GO:0007264	Small GTPase mediated signal transduction	1	1	1	0	0
GO:0006464	Cellular protein modification process	0	1	0	0	0
GO:0006508	Proteolysis	2	1	2	0	0
GO:0051726	Regulation of cell cycle	1	1	1	1	1
GO:0006955	Immune response	8	6	3	2	1
GO:0046907	Intracellular transport	1	1	0	0	0
GO:0007050	Cell cycle arrest	2	1	1	0	0
GO:0042981	Regulation of apoptotic process	3	2	2	1	1
GO:0023052	Signaling	0	1	0	0	0
GO:0019882	Antigen processing and presentation	6	5	3	3	1
GO:0006352	DNA-dependent transcription, initiation	1	1	0	0	0
GO:0006606	Protein import into nucleus	1	1	1	1	0
GO:0051056	Regulation of small GTPase mediated signal transduction	1	1	0	0	0
